# 2D and 3D-Organized Cardiac Cells Shows Differences in Cellular Morphology, Adhesion Junctions, Presence of Myofibrils and Protein Expression

**DOI:** 10.1371/journal.pone.0038147

**Published:** 2012-05-25

**Authors:** Carolina Pontes Soares, Victor Midlej, Maria Eduarda Weschollek de Oliveira, Marlene Benchimol, Manoel Luis Costa, Cláudia Mermelstein

**Affiliations:** 1 Instituto de Ciências Biomédicas, Universidade Federal do Rio de Janeiro, Rio de Janeiro, Brazil; 2 Laboratório de Ultraestrutura Celular, Universidade Santa Úrsula, Rio de Janeiro, Brazil; Dresden University of Technology, Germany

## Abstract

Cardiac cells are organized *in vivo* in a complex tridimensional structural organization that is crucial for heart function. While *in vitro* studies can reveal details about cardiac cell biology, usually cells are grown on simplified two-dimensional (2D) environments. To address these differences, we established a cardiac cell culture composed of both 2D and three-dimensional (3D)-organized cells. Our results shows significant differences between the two culture contexts in relation to the overall morphology of the cells, contraction ability, proliferation rate, presence of intercellular adhesion structures, organization of myofibrils, mitochondria morphology, endoplasmic reticulum contents, cytoskeletal filaments and extracellular matrix distribution, and expression of markers of cardiac differentiation. Cardiac cells grown in 2D-context displayed a flattened and well spread shape, were mostly isolated and their cytoplasm was filled with a large network of microfilaments and microtubules. In contrast, 3D-cells were smaller in size, were always in close contact with each other with several cellular junctions, and displayed a less conspicuous cytoskeletal network. 3D-cells had more mitochondria and myofibrils and these cells contract spontaneously more often than 2D-cells. On the other hand, endoplasmic reticulum membranes were present in higher amounts in 2D-cells when compared to 3D-cells. The expression of desmin, cadherin and alpha-actinin was higher in 3D-aggregates compared to 2D-spread cells. These findings indicate that the tridimensional environment in which the cardiac cells are grown influence several aspects of cardiac differentiation, including cell adhesion, cell shape, myofibril assembly, mitochondria contents and protein expression. We suggest that the use of this cardiac culture model, with 2D and 3D-context cells, could be useful for studies on the effects of different drugs, or growth factors, giving valuable information on the biological response of cells grown in different spatial organizations.

## Introduction

The heart is composed of contractile muscle cells and non-muscle cell types including fibroblasts and blood vessel cells. These cell types are organized in a complex tridimensional structural organization that is crucial for cardiac function and depends on autocrine, paracrine, and cell-cell interactions. Cardiac muscle cells, or cardiomyocytes, have the ability to contract due to the presence of a highly organized cytoskeleton. The cytoplasm of cardiomyocytes is filled with myofibrils, which are contractile fiber bundles composed of many functional units called sarcomeres [1]. The ends of myofibrils are anchored to the sarcolemma and the transmission of force developed by the contracting myofibrils is secured by highly specialized cell-cell junctions, the intercalated discs [2]. Two different intercellular adhesive junctions are found in the intercalated discs: adherens junctions and desmosomes, which anchor actin cytoskeleton and intermediate filaments, respectively, at the plasma membrane of adjoining cells, thereby provide mechanical attachment between the cells, and support the structural and functional integrity of the tissues [3]. Cardiac development is also regulated by the extracellular matrix, which forms a mesh of structural and signaling networks encapsulating and connecting the cells [4]. Any attempt to study cardiac cells must consider that a complex and intricate system of myofibrils connected to intercellular adhesion structures at the subsarcolemmal region of each cardiomyocyte is indispensable for cardiac function.

The controlled and simplified environment provided by culturing cardiac cells *in vitro*, has been important to identify molecules and mechanisms involved in their function and differentiation. Cardiomyocytes can be isolated from embryonic heart tissue from many different animal sources and placed into culture dishes at appropriate conditions. Different approaches have been used to grown cardiac cells *in vitro* [5]–[10]. These cells can rearrange their myofibrillar apparatus and even start to beat spontaneously again. Recent studies have reported that several cell types grown in three-dimensional (3D) contexts more closely resemble to *in vivo* cells both morphologically and in their molecular regulation [11]. This has been found to be particularly true for cardiomyocytes grown in 3D contexts [5], [12], as mechanical influences such as force and cell attachment are known to be involved in their maintenance and differentiation. It has been shown that the 3D topography where cardiac cells are grown significantly influences its morphology, myofibrillar protein stoichiometry and adhesion proteins expression [10]. An ideal cardiac cell culture should display important hallmarks of differentiated myocardium, such as highly organized sarcomeres, cellular junctions (adherens junctions, gap junctions, and desmosomes), and an extracellular matrix surrounding the cardiac cells.

While 3D cultures are more closely related to the *in vivo* situation, several important information have been gathered, and can be still obtained from 2D cultures. However, to our knowledge, a direct molecular and cellular comparison between 2D and 3D primary cultures of cardiac cells, growing together in the same conditions, has not been attempted. Therefore, in the present report, we describe a primary culture of chick cardiac cells that is composed of both 2D-cells and 3D-aggregates. In this culture system we were able to find significant differences between 2D- and 3D-cells in several parameters, including cell morphology, contraction ability, presence of adhesion structures, organization of myofibrils, mitochondria shape and contents, cytoskeletal filaments and extracellular matrix distribution and expression of proteins that are markers of cardiac differentiation. We suggest that this cardiac culture model could be useful for studies on the effects of different drugs, or growth factors, in 2D- versus 3D-contexts. A great deal of information, such as the analysis of cell proliferation, cell death, cell adhesion, cell migration, myofibril formation, protein expression and distribution, can be gathered from the two cell systems grown under the same culture media conditions.

Studies on different models of *in vitro* cardiac cultures are crucial for the understanding of the molecular and cellular basis of cardiac differentiation, and can be particularly important for cardiac tissue replacement therapy.

## Results and Discussion

Different cardiac cell culture protocols are found in the literature. We decided to study and compare directly 2D- and 3D-cardiac cells cultured together in many aspects, including their overall morphology, sub-cellular organization, ability to contract and the expression of markers of cardiac differentiation.

**Figure 1:**
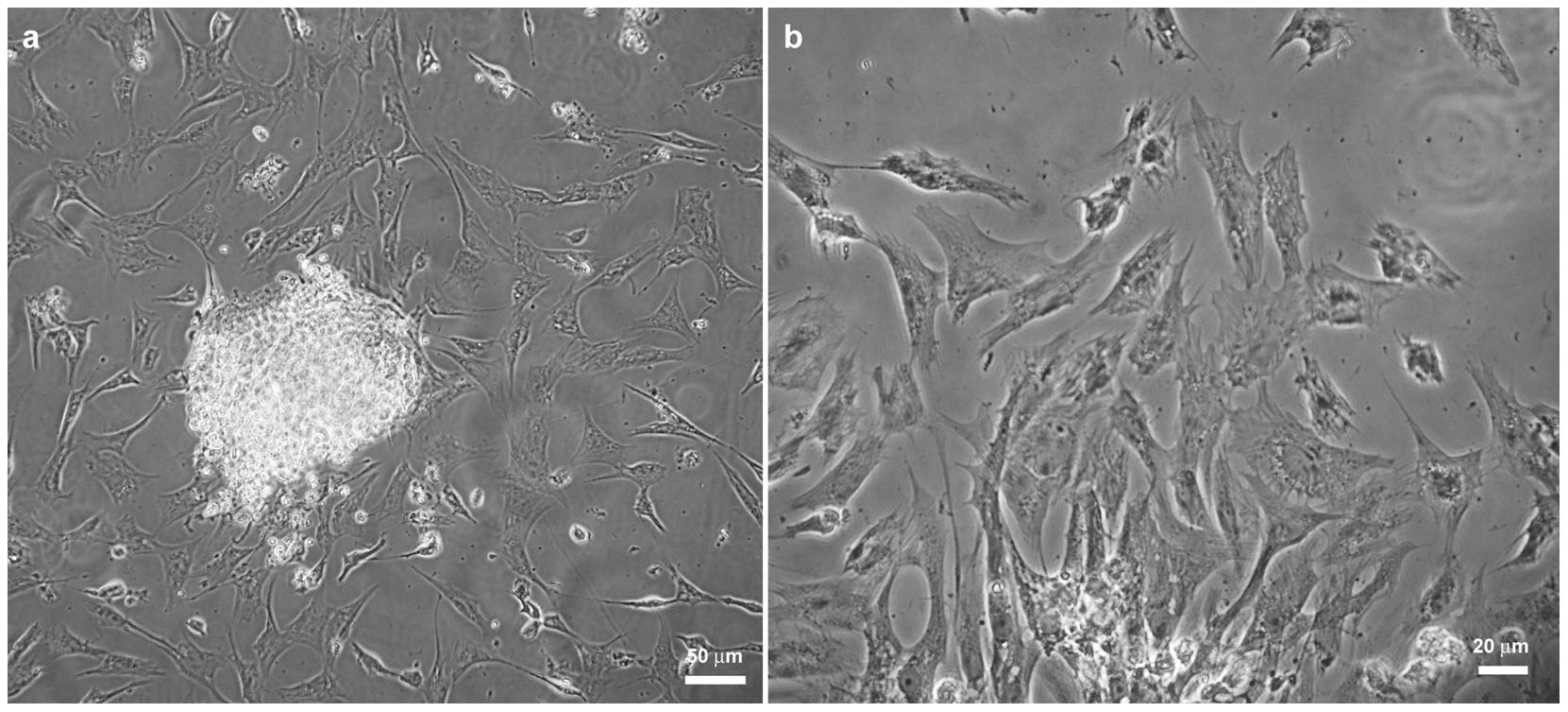
3D-cardiac cells form round shaped-structures while 2D-cardiac cells are flattened. 3D-cardiac cell aggregates and 2D-cardiac cells were isolated from hearts of 11-day-old chick embryos by mechanical and enzymatic dissociation and plated onto collagen-coated substrates. Phase-contrast microscopy of live cells shows 3D-organized cardiac aggregates (**a**) and well spread and flattened isolated cardiac cells (**a–b**).

**Figure 2 pone-0038147-g002:**
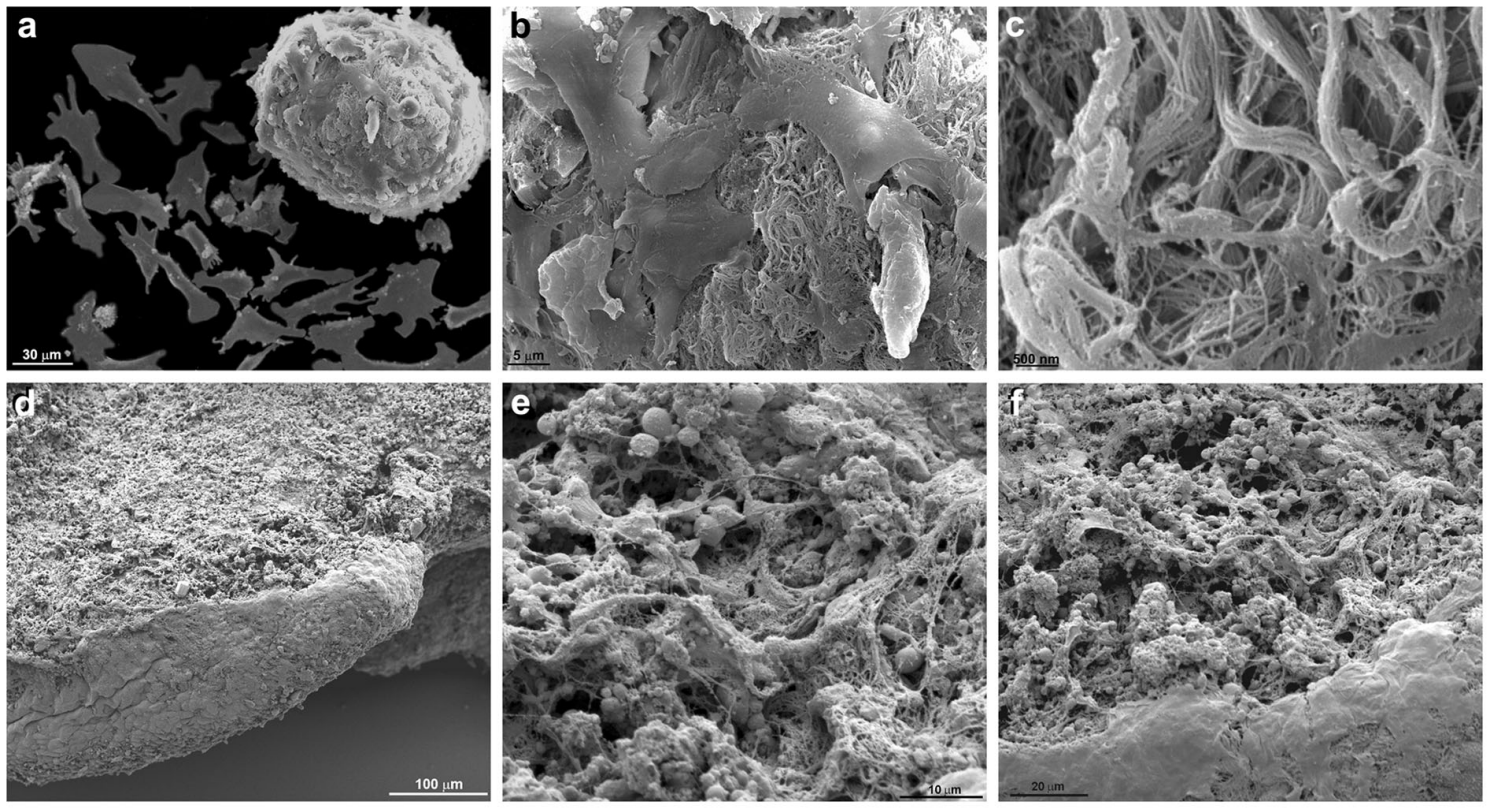
Scanning electron microscopy of 2D-cardiac cells and 3D-cardiac aggregates. 2D-cardiac cells are flattened and well-spread while a cardiac aggregate form a large cluster composed of cells in intimate contact (**a**). A large amount of extracellular matrix is seen connecting the cells in the 3-D aggregate (**b–c**). Note that the same aggregate is seen in detail after progressive higher magnifications (**a–c**). 3D-aggregates freshly isolated from chick embryo's hearts were also analyzed by SEM (**d–f**). Note the large amount of extracellular matrix found within the cardiac aggregates (**e–f**).

First, we analyzed the overall morphology of the cardiac cell cultures by phase contrast microscopy (**Figure 1**). Aggregates and dissociated cells were isolated and grown for 48 hours. Cultures displayed two clearly distinct groups of cells: 3D-organized cardiac aggregates (**Figure 1a**) and 2D-organized cardiac cells (**Figures 1a–b**). The 3D-aggregates were compact and round shaped-structures with ≈200 μm in diameter, composed of a large number of cells (in a density of ≈5000 cells/mm^2^). It has been described that one of the main challenges identified during *in vitro* cultivation of cardiac cells is the oxygen diffusion, coupled with a high oxygen demand by these cells, which limited the viable tissue thickness to ≈200 μm [13]. To obtain a reasonable oxygen influx within the aggregates, we tried to use cell clusters presenting about ≈200 μm or less. In contrast, the 2D-cardiac cells were well spread and flatten, showing a vast area of attachment to the substrate. It has been shown that cultured cardiomyocytes after spreading on 2D-culture dishes attain a flat and polygonal morphology, as opposed to the rod-shaped and bipolar cell morphology of differentiated cardiomyocytes found in the cardiac tissue [14].

**Figure 3:**
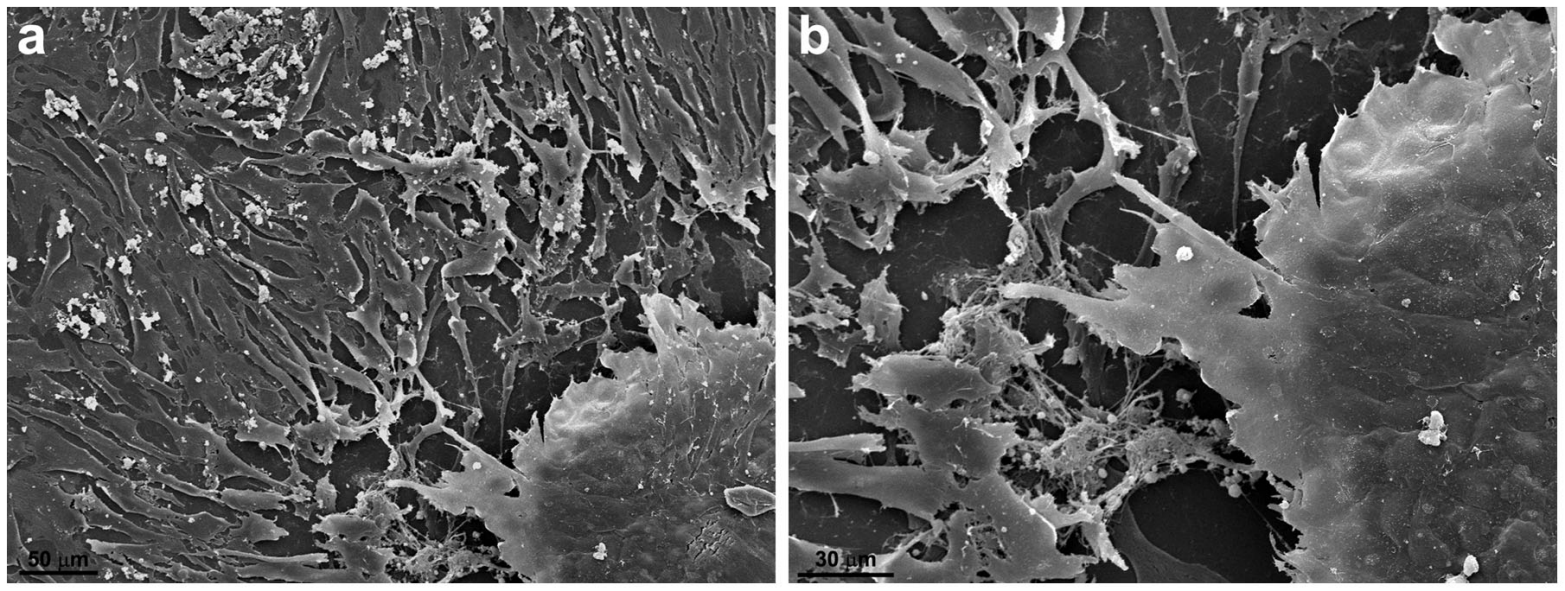
Scanning electron microscopy of 2D-cardiac cells and 3D-cardiac aggregates. In this image a large amount of isolated flattened cardiac cells almost fill the area around an aggregate. Connections and projections between the 2D-cells and the 3D-aggregate can be noted (**a–b**). Discrete lines indicating intimate contacts among the cells in the aggregate are clearly seen in image (**b**).

To further analyze the spatial cellular organization of the 2D- and 3D-cardiac cells, we studied the organization of both compartments by scanning electron microscopy (**Figures 2** **–** **3**). The 2D-cells were flattened and well-spread (**Figure 2a**), whereas the cardiac aggregates formed large clusters of cells in intimate contact with each other (**Figures 2a–c** **,** **3b**). In some areas of the cell cultures it was possible to see a large number of 2D-flattened cells occupying the whole area around the aggregates (**Figure 3a**). The cardiac aggregates varied in their thickness, and some aggregates were more flatten than others (for comparison see **Figures 2a** **,** **3b**). Several connections and projections between the 2D-cells and the 3D-cells were observed (**Figures 3a–b**). A large amount of extracellular matrix was seen connecting the cells in the 3D-aggregates (**Figures 2b–c**). Interestingly, it has been previously shown that 3D-cultures permitted cell extensions to become entangled with extracellular matrix fibrils, resulting in integrin-independent mechanical interactions that are not possible when cells attach to planar surfaces in 2D-cultures [15]. The authors suggest that cell-matrix entanglement represents a novel mechanism of cell anchorage that uniquely depends on the three-dimensional character of the matrix.

Since our results show a large amount of extracellular matrix connecting the cells in the 3D-aggregates (**Figures 2b–c**), we decided to analyze whether the 3D-cells were actively secreting extracellular matrix proteins or the matrix was already present in the embryonic cardiac tissue. We analyzed by scanning electron microscopy 3D-aggregates immediately after their isolation from chick embryo's hearts (**Figures 2e–f**) and compared with SEM of 3D-aggregates grown in culture for 48 hours (**Figures 2b–c**). 3D-aggregates analyzed immediately after their isolation from chick embryo's hearts display a large amount of extracellular matrix connecting the cells (**Figures 2e–f**), similar to 3D-aggregates grown in culture for 48 hours (**Figures 2b–c**). These results suggest that the structural arrangement and amount of extracellular matrix found in the chick heart tissue do not undergo major alterations during our *in vitro* culture conditions. Other experimental approaches will be are important to evaluate the extent of matrix protein turnover in our 3D culture system.

**Figure 4:**
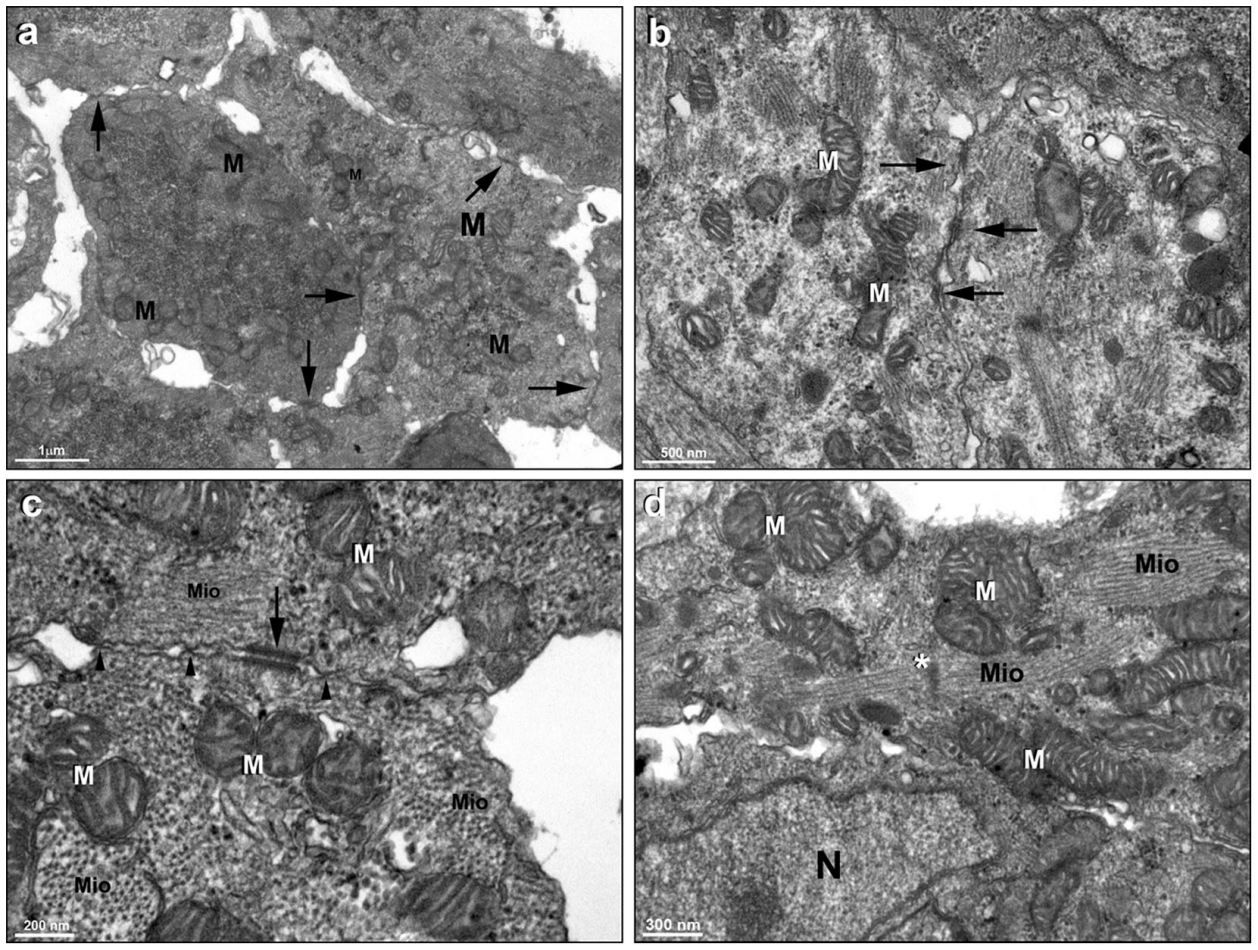
Transmission electron microscopy of 3D-cardiac aggregates. It is possible to notice a large number of cell-cell contacts (arrows), well-preserved mitochondria (M), and myofibrils (Mio) in a cardiac aggregate. Images (**b–c**) also show well-preserved mitochondria (M), organized myofibrils (Mio) and desmosomes (arrows), which are better visualized in image (**c**). Note in image (**c**) that some transversal sections of myofibrils (Mio) and other intercellular contacts are found (arrowheads). Image **d** shows myofibrils (Mio) with its Z lines (asterisks), as well several mitochondria (M) and a nucleus (N).

Because a number of differences in the overall organization between the 2D-cells and the 3D-aggregates was observed (**Figures 1** **–** [Fig pone-0038147-g002] **3**), we decided to analyze in more detail the ultrastructural organization of both groups of cells. Transmission electron microscopy of 3D-cardiac aggregates showed a large number of intercellular junctions (**Figures 4a–c**), organized myofibrils (**Figures 4c–d**) and well preserved mitochondria (**Figures 4a–d**). Desmosomes were found connecting neighboring cells (**Figure 4c**). The presence of a large number of cell-cell junctions in the 3D-cardiac aggregates is in accordance with the notion that in order to functionally contract, cardiac cells need to be in close contact with each other by means of adhesion sites, such as desmossomes or other adherens junctions types. It has been shown that cadherin/catenin adhesion complex can promote the extension of growing myofibrils towards the periphery of the cell, and also myofibril attachment at the cell-cell contact sites in cultured cardiac myocytes [16]. Our results show that the tridimensional organization of cardiac cells favors the establishment and maintenance of cellular junctions, mature myofibrils and a high number of mitochondria.

**Figure 5:**
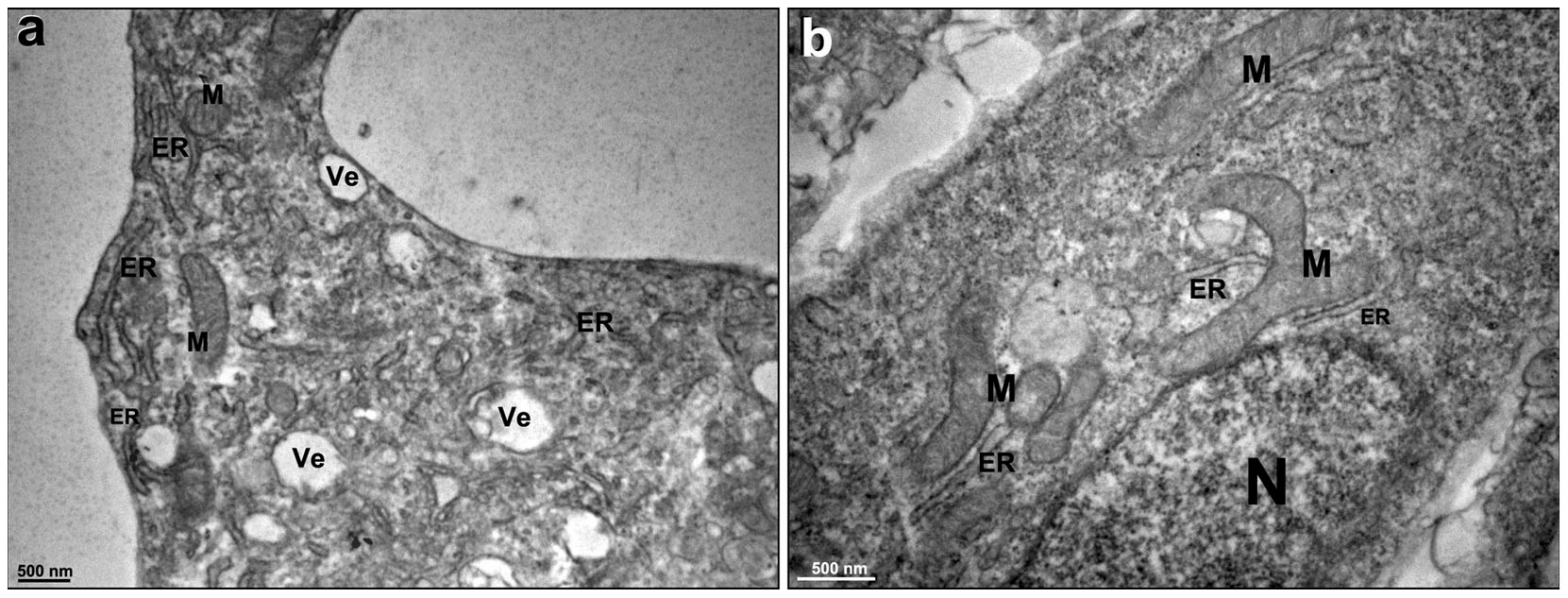
Transmission electron microscopy of 2D-cardiac cells. The 2D-cardiac cells are quite distinct from the 3D-cardiac cell aggregates (**a–b**), because they present several vesicles (Ve), a large endoplasmic reticulum (ER) and elongated mitochondria (M), some of which are branching, as seen in image (**b**). N, nucleus.

The ultrastructural organization of 2D-cultured cells was also analyzed using transmission electron microscopy. We observed that the 2D-cardiac cells were quite distinct from the 3-D cardiac cell aggregates: they presented more vesicles, a larger endoplasmic reticulum and prominent elongated mitochondria, some of which displayed a branched morphology (**Figures 5a–b**). The larger endoplasmic reticulum observed in the 2D-cells could be interpreted as a sign of increased protein synthesis and correlated with a high proliferation rate of the 2D-cells. The differences in mitochondria morphology found between 2D and 3D-cells could be related to differences in cell motility. It has been reported that during the process of cell spreading and movement, cells extend membrane lamellae and these structures are filled with microtubules and organelles such as mitochondria [17]. We can speculate that the 2D cells are rich in elongated mitochondria that are providing energy to the rapid spreading and movement of the cells.

**Figure 6:**
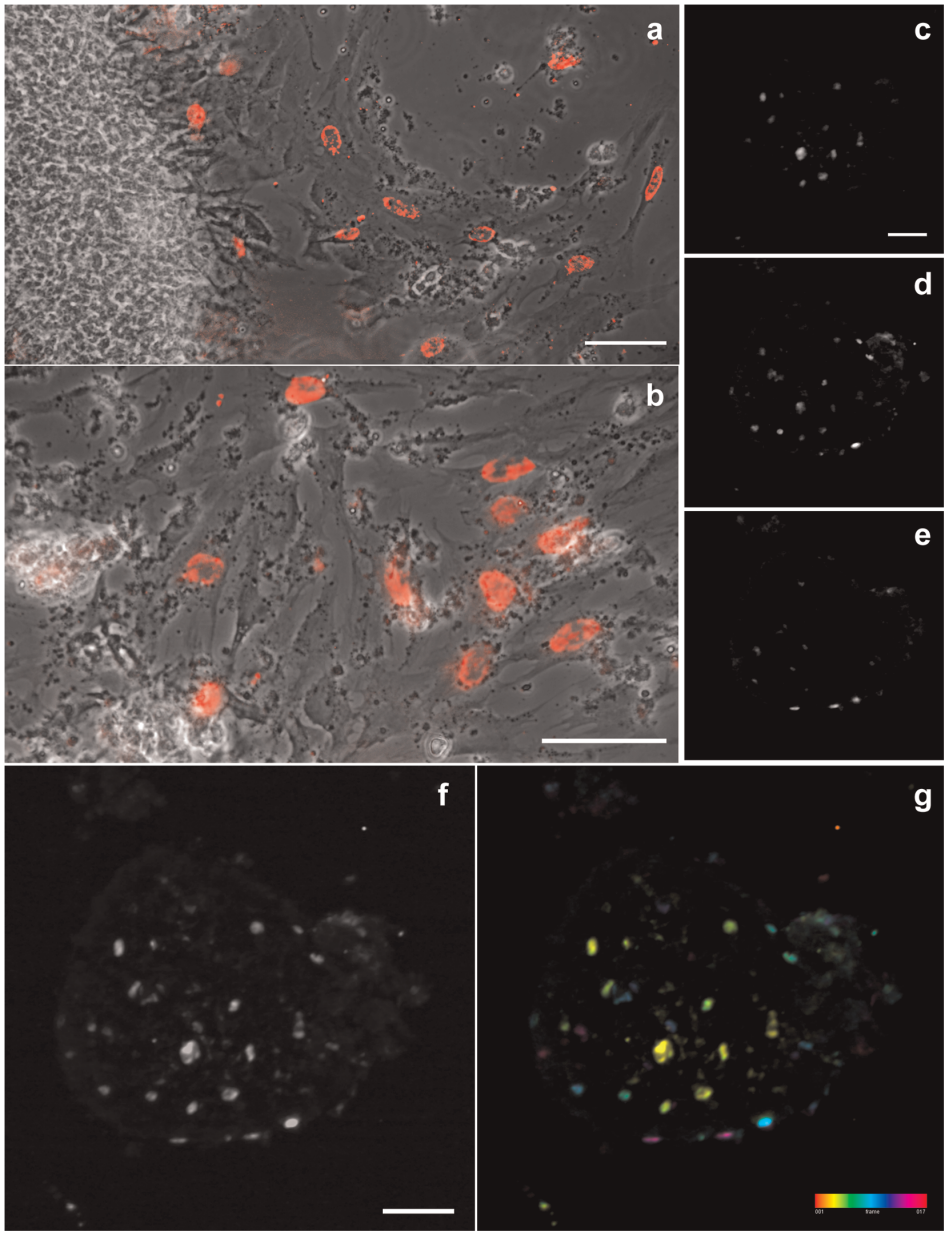
Proliferation rate of 2D and 3D-cardiac cells. 24-hs cardiac cell cultures were incubated with BrdU (3 ug/mL) and stained with anti-BrdU antibody. 2D-isolated cells were analyzed by phase contrast (**a–b**, gray) and conventional fluorescence microscopy (**a–b**, red), while 3D-aggregates were analyzed by confocal microscopy (**c–g**). Images (**a**, **b**) are different magnifications. BrdU-positive nuclei are found in both 2D and 3D-cells, but are more frequent in 2D-cells (**a–g**). Selected confocal slices, 10 µm apart, from bottom to top are shown in images (**c–e**). Note the difference in the distribution of BrdU positive cells along the aggregate Z-axis (**c–e**). All focal planes were merged in image (**f**), where it is possible to see the whole aggregate because of the faint fluorescence background. The relative position of all cells can be better visualized in the depth-color coded image shown in (**g**). The colored scale represents slice count, from bottom to top, each 2 µm apart. Scale bars in images (**a, b, c, f**) correspond to 50 µm.

In order to investigate the proliferation rate of 2D and 3D-cells, we decided to analyze the incorporation of BrdU in these cardiac cell cultures (**Figure 6**). 2D-isolated cells were analyzed by phase contrast and conventional fluorescence microscopy (**Figures 6a–b**), while 3D-aggregates were analyzed by confocal microscopy (**Figures 6c–g**). BrdU-positive nuclei were found in both 2D and 3D-cells, but were more frequent in 2D-cells than in 3D-aggregates. We estimated 23% of BrdU-positive cells in 3D-aggregates compared to 35% in 2D-cells. Although cultured heart cells do undergo mitosis, there is no general agreement on whether the mitotic state affects the state of differentiation [12], [18]. Some investigators suggest that dividing cells are not in a highly differentiated state. That could explain the less differentiated and more proliferative state of the 2D-cells as compared to the 3D-cells.

**Figure 7:**
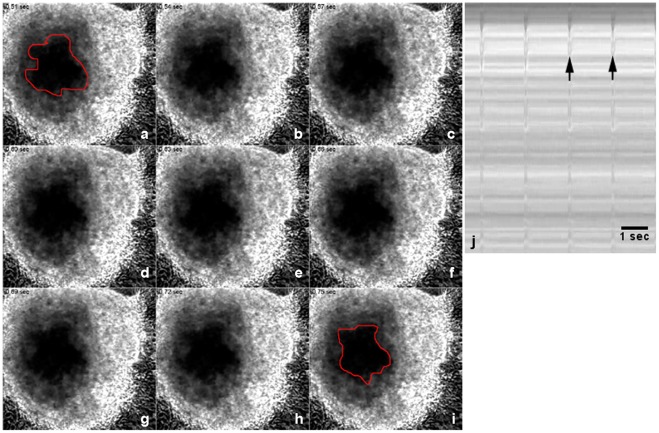
3D-cardiac aggregates spontaneously contract in culture. 3D-cardiac aggregates and 2D-cardiac cells were gown for 48 hours and then live cultures were analyzed under phase contrast microscopy. The recorded images shown in images (**a–I**) have an interval of 0.03 seconds. Note that the central area marked in red is larger in image (**a**) than in image (**i**), showing the reduction in size of the aggregate and therefore their contraction. A temporal profile corresponding to images (**a–I**) is shown in (**j**)**,** and arrows point to contraction events.

We also analyze the functional ability of the *in vitro* cardiac cells to contract using phase contrast videomicroscopy. 3D-cardiac aggregates were capable of spontaneous contractions (**Figure 7** **– Movie S1**), whereas only a few 2D-cardiac cells fairly exhibited contractions. Temporal analysis showed that cardiac aggregates contracted at a rate of 102 beats per minute with the duration of 0.5 seconds (**Figure 7j**). These contraction results are supported by the data presented in **Figure 4** **,** which shows in the 3D-cells by transmission electron microscopy a larger number of intercellular junctions, organized myofibrils, extracellular matrix deposition and abundance of mitochondria. Previous studies have established that contraction of cardiac muscle cells is dependent on the presence of striated myofibrils, their linkage to adhesion sites at the sarcolemma, and the presence of a significant number of ATP-producing mitochondria [19], [20]. Furthermore, it has been shown that cardiac cell density and extracellular matrix composition affect the synchronized contraction of cardiomyocytes grown in 3D-organized structures [9]. We can speculate that the density of cardiomyocytes and the amount and composition of extracellular matrix present in the 3D-aggregates meet these requirements for contraction. Our results are also in accordance with the work of Renauld and Sperelakis [21] that reported that small chick heart fragments (of about 1 mm^3^) tend to retain their highly differentiated electrical properties when cultured.

**Figure 8:**
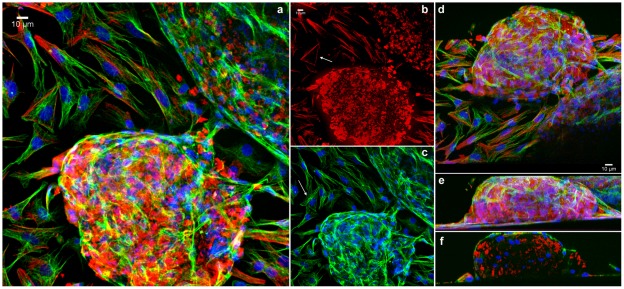
Analysis of the distribution of the cytoskeleton in 2D- and 3D-cardiac cultures. Cells were grown for 48 hours, fixed with 4% paraformaldehyde and triple-stained with Texas red-phalloidin (red), mouse monoclonal anti-α-tubulin antibody (green), and the DNA-specific probe DAPI (blue). Cells were analyzed in a Leica laser scanning confocal microscope. Different optical focal planes of the cells were acquired and projected in order to show both the 2D-cells and the 3D-aggregates (**a**). The same stack in **a** can be seen with the F-actin stain only, where 2D-cells display well organized stress fibers (arrow, **b**) whereas no detectable stress fibers are seen in the 3D-aggregates (**b**). Again, in the same stack in (**a**, **b**), microtubules and nuclei were superposed to show well spread microtubules in 2D-cells (arrow), whereas only a few cells in the surface of the aggregates display organized microtubules (**a, c**). Note MTOCs close to the nuclei in 2D-cells. The same stack can be seen in a tilted projection in (**d**), showing the relationship of 2D and 3D cells. The same stack can be seen in a lateral view, showing the volume of the aggregate and the connection between 2D and 3D-cells (**e**). Lastly, the same stack is shown in a transverse section of a lateral projection, showing the distribution of microtubules, microfilaments and nuclei inside the aggregate (**f**).

**Figure 9:**
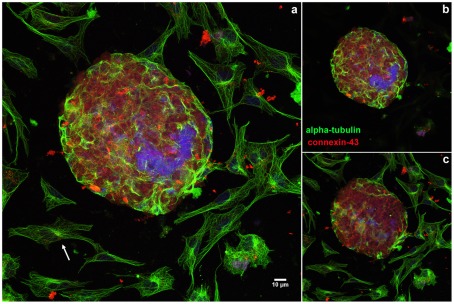
Comparison of the distribution of microtubules in 2D- and 3D-cardiac cultures. Cells were grown for 48 hours, fixed with 4% paraformaldehyde and triple-stained with polyclonal anti-connexin-43 antibody (red), mouse monoclonal anti-α-tubulin antibody (green), and the DNA-specific probe DAPI (blue). Cells were analyzed in a Leica laser scanning confocal microscope. Different optical focal planes of the cells were acquired and projected in order to show both the 2D-cells and the 3D-aggregates. 2D-cells display an extensive microtubular network (arrow, **a**), whereas only a few cells in the surface of the aggregates display organized microtubules. An intense labeling of connexin 43 is only seen in the 3D-aggregates (**a**). To highlight the differences between the 2D and 3D-cells, we projected the slices corresponding to the 3D-aggregate only in image (**b**) and the slices corresponding to the 2D-cells only in image (**c**).

To compare the spatial distribution of the main cytoskeletal elements, microfilaments and microtubules, between 2D- and 3D-aggregates, we used laser-scanning confocal microscopy. It has been shown that the microfilament and microtubule organization in cells are dynamically modulated by physical and biological information from the environment where the cells are grown. For example, actin filaments are typically organized *in vitro* in stress fibers, which are contractile actomyosin bundles essential for cell adhesion to the substratum. Stress fibers are typically associated at both their extremities to focal adhesions and their appearance and stability is influenced by the effective stiffness or rigidity of a flat substrate [22], [23]. To study the organization of the cytoskeleton in the cardiac cell cultures, cells were triple-stained using Phalloidin, anti-α-tubulin antibody and DAPI (**Figure 8** **–** **Movie S2**); or triple-labelled using anti-α-tubulin and anti-connexin 43 antibodies, followed by DAPI staining (**Figure 9**). Cells were analyzed in a laser confocal microscope where different optical focal planes of the cells were acquired and projected in order to show both the 2D-cells (which are closer to the substrate) and the 3D-aggregates (which are thicker). Significant differences were found in the distribution of microfilaments and microtubules between the well spread 2D-cells and the 3D-aggregates. The 2D-cells displayed well organized stress fibers (**Figure 8b** **, arrow**) and an extensive microtubular network (**Figures 8c** **,** **9a**, arrows), whereas no detectable stress fibers were seen in the 3D-aggregates (**Figures 8a–b**) and only a few cells in the surface of the aggregates displayed organized microtubules (**Figures 8a–c** **,** **9a–c**). Only in 2D-cells we found microtubules organizing centers (MTOCs) in close proximity to the nuclei (**Figure 8**). Connexin 43 was observed in the 3D-aggregates whereas no detectable labeling of this protein was detected in the 2D-cells (**Figure 9a**). It has been shown that stress fibers can rarely be seen in tissues, except under conditions of wound repair and fibrosis [24]; conversely these F-actin based-structures are often seen in 2D-cultures. Motlagh and colleagues [10] described non-striated actin cables in cardiomyocytes plated on flat 2D surfaces as opposed to the presence of striated actin structures present in 3D surfaces. Further, it has been shown that in order to adapt to the *in vitro* 2D-situation cardiomyocytes have to undergo a complex dedifferentiation redifferentiation process, which seems to be dependent on an intact microtubule array [14]. The data presented here show that differences in the spatial organization of cardiac cells result in a different organization of the microfilaments and microtubular cytoskeleton.

**Figure 10:**
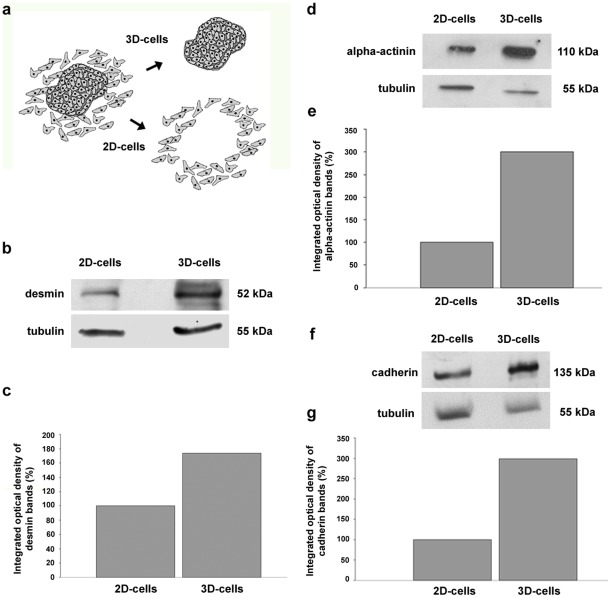
Expression of desmin, alpha-actinin and cadherin is higher in 3D-aggregates than in 2D-cells. 3D-aggregates and 2D-cardiac cells were gown for 48 hours and then collected separately as two different samples (**a**). Samples were analyzed by SDS-PAGE followed by Western blots using antibodies against desmin, sarcomeric alpha-actinin and cadherin (**b, d, f**). α-tubulin reactivity of the same samples was used to normalize sample loading. Quantification of protein bands revealed that the expression of all three proteins was higher in 3D-aggregates than in 2D-cells (**c, e, g**).

Because differences in the presence of cellular junctions and myofibrils were found in the 2D- and 3D-cardiac cells, we decided to follow the quantitative expression of markers of cardiac differentiation: desmin, sarcomeric alpha-actinin and cadherin. The transmembrane protein cadherin is essential of cardiomyocyte intercellular adhesions [25], alpha-actinin is a key component of the myobrillar apparatus and desmin is a muscle-specific intermediate filament that participates in the structural linkage between the myofibrils and the sarcolemma [26]. Quantification of immunoblotting (**Figure 10a**) revealed that relative expression of desmin, sarcomeric alpha-actinin and cadherin was higher in 3D-aggregates than in 2D-cells (**Figures 10b–g**). These results are in accordance with the data presented in **Figure 4**, which shows a large number of cell-cell junctions and myofibrils in the 3D-cardiac aggregates. Cadherin is the main component of the cardiac cell junctions and alpha-actinin and desmin are present in myofibrils. The higher desmin expression found in the 3D-aggregates could be related to a higher number of cardiomyocytes present in this compartment in comparison to the 2D region.

What is the cell type composition of the 2D and 3D-compartments? Recently, we have shown that both 2D-isolated cardiac cells and the 3D-cardiac cells express the muscle specific proteins desmin, caveolin-3 and sarcomeric tropomyosin [7], thus characterizing the presence of cardiomyocytes in both 2D and 3D regions. While we could not define the relative cellular composition of the two compartments, it is clear that they both have cardiomyocytes and other cell types, probably fibroblasts.

Our data show that the *in vitro* 3D-chick cardiac cells more closely resembles the *in vivo* heart tissue than the *in vitro* 2D-chick cardiac cells. Some characteristics of the chick 3D-cardiac aggregates are similar to engineered 3D-cardiac tissue constructs and both models offers perspectives for basic cardiovascular research and for tissue replacement therapy. Two main approaches for cardiac tissue replacement therapy have been described in the last two decades: the use of isolated cells and the use of *in vitro*-engineered tissue equivalents [8], [9], [10]. The main difference from our model of 3D-aggregate cells and others is that we use 3D-cells that are in fact small heart fragments (with ≈200 μm in thickness) instead of heart tissue reconstituted from isolated cardiac cells. This might explain the high number of sarcomeric structures, specialized cell-cell junctions, mitochondria and the abundance of extracellular matrix that we found in the 3D-aggregates. Reconstitution of isolated cardiac cells into 3D-structures usually involves the disassembly of myofibrils prior to their reassembly. In contrast, we hypothesize that the cardiac structures are better preserved in the small heart fragments because they do not seem to undergo this disassembly-reassembly step.

In summary, we studied 2D and 3D-cardiac cells grown together under the same culture, and we were able to establish key molecular and cellular parameters that we used to compare them. Our results showed differences between 2D and 3D-cells in intercellular adhesions, cell motility, proliferation rate, deposition of extracellular matrix, mitochondrial morphology, formation of sarcomeric structures, ability to spontaneous contraction, and expression of key markers of cardiac differentiation. These differences are probably not only due to the type of the cells, but to their environment. Moreover, we showed that cells could move from the 3D and 2D-compartments and possibly change their phenotype, reinforcing the notion that cardiac cells respond in a very dynamic way to the surrounding environment. Thus, we suggest that a co-culture system containing both 2D- and 3D-primary cardiac cells is a useful model for studies focusing the molecular and cellular responses of cardiac cells to different molecules and drugs.

## Materials and Methods

### Antibodies and fluorescent probes

The DNA-binding probe 4,6-diamidino-2-phenylindole dihydrochloride (DAPI) and the F-actin probe Texas red-Phalloidin were purchased from Molecular Probes (Eugene, OR, USA). Mouse monoclonal anti-BrdU, mouse monoclonal anti-α-tubulin (clone DM 1A), mouse monoclonal anti-sarcomeric alpha-actinin (clone EA53), rabbit polyclonal anti-desmin, rabbit polyclonal anti-connexin 43, and rabbit polyclonal anti-pan-cadherin antibodies were purchased from Sigma Chemical Co. (St. Louis, MO, USA). 488 and 456 Alexa fluor-conjugated goat anti-rabbit and goat anti-mouse immunoglobulin G (IgG) antibodies were purchased from Molecular Probes (USA). Peroxidase-conjugated goat anti-mouse and anti-rabbit antibodies and peroxidase-conjugated mouse anti-goat antibody were purchased from Santa Cruz (USA).

### 2D and 3D primary chick cardiac cell cultures

This study using chick embryos was approved by the Ethics Committee for Animal Care and Use in Scientific Research from the Federal University of Rio de Janeiro and received the approval number: DAHEICB 004. Primary chick cardiac cultures were prepared according to Pontes Soares and colleagues [7]. All cell culture reagents were purchased from Invitrogen (São Paulo, Brazil). Primary cultures of cardiac cells were prepared from 11-day-old chick embryos (Granja Tolomei, Brazil). Fragments of cardiac muscle were incubated at 37°C for 10 min in calcium-magnesium-free solution (CMF) containing 0.2% trypsin. Then, the supernatant was gently removed and discarded. The fragments of cardiac muscle were further incubated five times at 37°C for 5 min in CMF containing 0.2% trypsin. After each trypsin incubation, the supernatant was gently removed and placed into chilled plating medium (5% fetal bovine serum, 1% L-glutamine, and 1% penicillin-streptomycin in Minimum Essential Medium – MEM). Following the final trypsin incubation, the remaining tissue fragments were mechanically dispersed by gently pipetting, and the resulting suspension was placed into the chilled plating medium containing previously dispersed cells. The suspension containing isolated cells and tissue fragments was centrifuged, and the pellet was dispersed by repeated pipetting in plating medium. Cells and tissue fragments were plated on 22 mm Aclar plastic coverslips (Pro-Plastics Inc., Linden, NJ, USA) previously coated with rat-tail collagen, and placed in 35 mm plastic culture dishes (2 embryonic hearts per dish). Cells were grown under humidified 5% CO_2_ atmosphere at 37°C. The day after plating, the nutrient medium was replaced with growth medium (5% fetal bovine serum and 1% penicillin–streptomycin in MEM). Elimination of L-glutamine from the medium has been shown to facilitate the culture of cardiac cells by reducing the proliferation of cardiac fibroblasts. The growth medium was replaced daily.

### BrdU incorporation assay

5-bromo-2′-deoxyuridine (BrdU, Sigma, 3 ug/mL) was added to cardiac cells (with 24 hours) for 1 hour. Cells were fixed with 4% paraformaldehyde in PBS for 15 minutes at room temperature and subsequently washed twice with PBS at 37°C for 15 minutes and once with deionized water at 37°C for 10 minutes. Cells were incubated with 2 N HCl at 37°C for 30 minutes and washed twice with 0.1 M Borate buffer (pH 8.5) at 37°C for 5 minutes each and once with PBS. Cells were then stained with anti-BrdU primary antibody. After incubation, cells were washed for 30 min and incubated with Alexa Fluor 546-conjugated secondary antibody and analyzed by phase contrast microscopy, conventional fluorescence microscopy (Axiovert 100 microscope with phase-contrast optics, Carl Zeiss, Germany) and confocal fluorescence microscopy (Disk Spinning Unit confocal microscope, DSU, Olympus, Japan). To evaluate the proportion of dividing cells in the culture, we counted the number of BrdU-positive cells per area in the aggregates (1122±477 BrdU-positive nuclei/mm^2^). We also counted the number of nuclei per area using DAPI (4970±1621 nuclei/mm^2^). We then compared both values to calculate the proportion of replicative cells. For the quantification of proliferative 2D-cells, we directly compared the BrdU labeling with the phase contrast image.

### Immunofluorescence microscopy and digital image acquisition

Cultured cells were rinsed with PBS and fixed with 4% paraformaldehyde in PBS for 10 min at room temperature. Then, cells were permeabilized with 0.5% Triton-X 100 in PBS three times for 10 min. The same solution was used for all subsequent washing steps. Cells were incubated with primary antibodies for 1 h at 37°C. After incubation, cells were washed for 30 min and incubated with Alexa Fluor 488- or Alexa Fluor 546-conjugated secondary antibodies for 1 h at 37°C. Nuclei were labeled with DAPI (0.1 μg/ml in 0.9% NaCl). Cells were washed for 5 min with 0.9% NaCl, and specimens were mounted in glycerol containing, by weight, 5% n-propyl gallate, 0.25% DABCO (1,4–diazabicyclo(2,2,2)octane), and 0.0025% para-phenylenediamine (all from Sigma). Cardiac cultures were analyzed in a laser scanning confocal microscope (TCS SP5 AOBS, Leica, Japan). Image processing and stack projections were performed using Fiji software (based on ImageJ, http://imageJ.nih.gov/ij/). Control experiments with no primary antibodies showed only a faint background staining (data not shown). Live cultured cells grown in collagen-coated Aclar coverslips were examined in an Axiovert 100 microscope with phase-contrast optics (Carl Zeiss, Germany), and images were acquired with an Olympus DP72 digital camera (Olympus, Japan).

### Contraction measurements

3D-aggregates and 2D-cardiac cells were gown for 48 h and then live cultures were analyzed under phase contrast microscopy in an Axiovert 100 microscope with phase-contrast optics (Carl Zeiss, Germany). Images were recorded with an Olympus DP72 digital camera (Olympus, Japan) and a temporal profile of contraction was generated using Fiji software.

### Polyacrylamide gel electrophoresis and immunoblotting

Cell cultures were quickly washed in ice-cold PBS, and all the 3D-aggregates of each culture dish were transferred to an eppendorf tube by using a small needle. The remaining cells, which correspond to the 2D-cells, were harvested and collected to another eppendorf tube. Both samples (2D- and 3D-cells) were treated with sample buffer (4% SDS, 20% glycerol, 10% 2-mercaptoethanol, 125 mM Tris-HCl pH 6.8) and boiled for 10 min. Samples were loaded in 10% SDS-polyacrylamide gel electrophoresis. Proteins were transferred overnight at 50 Volts to PVDF membranes. The proteins immobilized on the membranes were immediately blocked for 1-h at room temperature with a solution of 5% nonfat dry milk in Tris buffered saline-Tween 20 (0.001%) (TBS-T). Then, the membranes were incubated with primary antibodies (diluted in TBS-T). After five washes in TBS-T (three minutes each), the membranes were incubated either with an anti-rabbit, anti-mouse, or anti-goat peroxidase conjugated antibody (Amersham, dilution 1:10,000 in TBS-T), and washed again as described above. The bands were observed using the ECL plus Western Blotting Detection System (Amersham, USA). To check sample loading, duplicate membranes were incubated with a mouse monoclonal anti-α-tubulin antibody (Sigma, dilution 1:5000 in TBS-T-milk). After five washes in TBS-T (three minutes each), membranes were incubated with anti-mouse peroxidase conjugated antibody (Amersham, dilution 1:10,000 in TBS-T) and developed as described above.

### Scanning electron microscopy (SEM)

Cardiac cell cultures have had the culture medium removed and were quickly washed in warm PBS and next, they were fixed *in situ* with 2.5% glutaraldehyde in 0.1 M sodium cacodylate buffer, pH 7.2, overnight. Afterwards, the cells were washed in PBS, post-fixed for 40 min in 1% OsO_4_ diluted in 0.1 M sodium cacodylate buffer, pH 7.2, dehydrated in crescent grades of ethanol, critical point dried with liquid/gas CO_2_, and sputter-coated with 15 nm-thick gold-palladium. The samples were examined in a JEOL 5800 scanning electron microscope using acceleration voltage of 25 KV.

### Transmission electron microscopy (TEM)

#### For 3D-aggregates

Cardiac cell cultures were collect as described above for SEM, fixed overnight at room temperature in 2.5% (v/v) glutaraldehyde in 0.1 M cacodylate buffer, pH 7.2. Afterwards, the cells were washed three times in PBS and post-fixed for 40 min in 1% OsO4 in 0.1 M cacodylate buffer containing 5 mM CaCl_2_ and 0.8% potassium ferricyanide. The cells were dehydrated in acetone and embedded in Epon. Two procedures were tested: (1) aggregates cardiac cells were removed from the flasks using a rubber policeman and after spin down the pellet was routinely processed for TEM as described above. In a second procedure (2), the aggregates were fixed and processed in situ in the same Falcon flask were the cells were grown. All the subsequent steps were performed with the cells still in the flask. After embedding, a thin layer of the embedded cells was cut and added to a new empty Epon bloc. Ultra-thin sections with 70–90 nm thick were harvested on 300-mesh copper grids, stained with 5% uranyl acetate and 1% lead citrate, and then observed with a JEOL 1210 transmission electron microscope.

#### For 2D-cell

The cardiac aggregates were manually removed from the cell culture and the remaining cells were submitted to fixation, post-fixation, dehydration and embedding in the Petri dish using the same procedure described above for the 3D-aggregates.

## Supporting Information

Movie S1 **Spontaneous contraction of a 3D-aggregate.** Real-time phase contrast videomicroscopy of a 3D-cell aggregate beating. Selected frames from this movie can be seen in **Figure 7** **.**
(AVI)

Movie S2 **Comparison of the distribution of microtubules and microfilaments in 2D- and 3D-cardiac cultures.** Three-dimensional projection of confocal stack stained for α-tubulin (green), F-actin (red) and nuclei (blue). Two 3D aggregates and several 2D cells can be seen. Note the volume of the aggregate and the relationship between 2D and 3D cells. This is the same stack shown in **Figure 8**.
(AVI)

## References

[1] Gautel M (2011). The sarcomeric cytoskeleton: who picks up the strain?. Curr Opin Cell Biol.

[2] Sheikh F, Ross RS, Chen J (2009). Cell-cell connection to cardiac disease.. Trends Cardiovasc Med.

[3] Noorman M, van der Heyden MA, van Veen TA, Cox MG, Hauer RN (2009). Cardiac cell-cell junctions in health and disease: Electrical versus mechanical coupling.. Mol Cell Cardiol.

[4] Kresh JY, Chopra A (2011). Intercellular and extracellular mechanotransduction in cardiac myocytes.. Pflugers Arch.

[5] Akins RE, Rockwood D, Robinson KG, Sandusky D, Rabolt J (2010). Three-dimensional culture alters primary cardiac cell phenotype.. Tissue Eng Part A.

[6] Decker ML, Behnke-Barclay M, Cook MG, La Pres JJ, Clark WA (1991). Cell shape and organization of the contractile apparatus in cultured adult cardiac myocytes.. Mol Cell Cardiol.

[7] Pontes Soares C, Portilho DM, da Silva Sampaio L, Einicker-Lamas M, Morales MM (2010). Membrane cholesterol depletion by methyl-beta-cyclodextrin enhances the expression of cardiac differentiation markers.. Cells Tissues Organs.

[8] Zimmermann WH, Schneiderbanger K, Schubert P, Didié M, Münzel F (2002). Tissue engineering of a differentiated cardiac muscle construct.. Circ Res.

[9] Shapira-Schweitzer K, Seliktar D (2007). Matrix stiffness affects spontaneous contraction of cardiomyocytes cultured within a PEGylated fibrinogen biomaterial.. Acta Biomater.

[10] Motlagh D, Senyo SE, Desai TA, Russell B (2003). Microtextured substrata alter gene expression, protein localization and the shape of cardiac myocytes.. Biomaterials.

[11] Elliott NT, Yuan F (2010). A review of three-dimensional *in vitro* tissue models for drug discovery and transport studies.. J Pharm Sci.

[12] Sperelakis N (1978). Cultured heart cell reaggregate model for studying cardiac toxicology.. Environ Health Perspect.

[13] Radisic M, Malda J, Epping E, Geng W, Langer R (2006). Oxygen gradients correlate with cell density and cell viability in engineered cardiac tissue.. Biotechnol Bioeng.

[14] Ehler E, Perriard JC (2000). Cardiomyocyte cytoskeleton and myofibrillogenesis in healthy and diseased heart.. Heart Fail Rev.

[15] Jiang H, Grinnell F (2005). Cell-matrix entanglement and mechanical anchorage of fibroblasts in three-dimensional collagen matrices.. Mol Biol Cell.

[16] Goncharova EJ, Kam Z, Geiger B (1992). The involvement of adherens junction components in myofibrillogenesis in cultured cardiac myocytes.. Development.

[17] Bereiter-Hahn J, Lück M, Miebach T, Stelzer HK, Vöth M (1990). Spreading of trypsinized cells: cytoskeletal dynamics and energy requirements.. J Cell Sci.

[18] Claycomb WC (1992). Control of cardiac muscle cell division.. Trends Cardiovasc Med.

[19] Clark KA, McElhinny AS, Berckerle MC, Gregorio, CC (2002). Striated muscle cytoarchitecture: an intricate web of form and function.. Annu Rev Cell Dev Biol.

[20] Kaasik A, Kuum M, Joubert F, Wilding J, Ventura-Clapier R (2010). Mitochondria as a source of mechanical signals in cardiomyocytes.. Cardiovas Res.

[21] Renaud J-F, Sperelakis N (1976). Electrophysiological properties of chick embryonic hearts grafted and organ-cultured *in vitro*.. J Mol Cell Cardiol.

[22] Engler AJ, Sen S, Sweeney HL, Discher DE (2006). Matrix elasticity directs stem cell lineage specification.. Cell.

[23] Saez A, Ghibaudo M, Buguin A, Silberzan P, Ladoux B (2007). Rigidity-driven growth and migration of epithelial cells on microstructured anisotropic substrates.. Proc Natl Acad Sci U S A.

[24] Byers HR, White GE, Fujiwara K (1984). Organization and function of stress fibers in cells *in vitro* and *in situ*. A review.. Cell Muscle Motil.

[25] Zuppinger C, Eppenberger-Eberhardt M, Eppenberger HM (2000). N-cadherin: structure, function and importance in the formation of new intercalated disc-like cell contacts in cardiomyocytes.. Heart Fail Rev.

[26] Costa ML, Escaleira RC, Cataldo A, Oliveira F, Mermelstein CS (2004). Desmin: molecular interactions and putative functions of the muscle intermediate filament protein.. Braz J Med Biol Res.

